# Diversity and antibiotic sensitivity of uropathogens from a regional hospital in Rwanda

**DOI:** 10.4314/ahs.v24i3.4

**Published:** 2024-09

**Authors:** Charles O Odongo, Jean Baptiste Niyibizi, Ezechiel Bizimana, Irènèe Nshimiyimana, Deogratias Ruhangaza

**Affiliations:** 1 Division of Basic Medical Sciences, School of Medicine, University of Global Health Equity, P.O.Box 6955, Kigali-Butaro, Rwanda; 2 Pathology Department, Butaro District Hospital, Burera District, Northern Province, Rwanda

**Keywords:** Diversity and antibiotic sensitivity, uropathogens, Rwanda

## Abstract

**Background:**

Urinary tract infections are a common cause for antibiotic consumption. Empirical treatment is common for community-acquired infections owing to predictability of pathogens.

**Objectives:**

Describe sensitivity profiles of uropathogens at a regional hospital in Rwanda.

**Methods:**

Hospital-based cross-sectional study in which clean-catch urine samples were analyzed. A two-stage process involving dipstick urinalysis and urine culture was used to identify true infections. The Kirby-Bauer disc diffusion method was used for antibiotic sensitivity analysis as per EUCAST guidelines.

**Results:**

Of 198 samples studied, 107 met criterion for UTI with 94 yielding significant growth. Klebsiella pneumoniae (35%), and Escherichia coli (32%) were the most common organisms isolated. Others included Staphylococcal species, Proteus spp, Enterobacter spp and Salmonella. Very high resistance frequencies (above 70%) were observed against amoxycillin, cotrimoxazole, trimethoprim, aztreonam and fosfomycin. Resistance to fluoroquinolones, aminoglycosides and cephalosporins varied between 40-60%. High levels of cross resistance were observed for drugs of the same class. Resistance to the potentiated penicillins, meropenem, cefoxitin and nitrofurantoin were less than 30%. Piperacillintazo bactum performed best with 91% sensitivity against all organisms tested.

**Conclusion:**

High levels of resistance were observed for most antibiotics studied. For empirical treatment of community-acquired UTI in our setting, nitrofurantoin remains effective while ciprofloxacin is not.

## Introduction

Urinary tract infections (UTI) are among the most common bacterial infections encountered in practice. Clinical features range from dysuria in uncomplicated cystitis to abdominal or flank pain coupled with fever, nausea and vomiting, seen in acute pyelonephritis^1^. In the United States where data collation is comparatively advanced, UTI account for approximately 15% of all antibiotic prescriptions^2^. In general, females are more prone to UTI than males, a phenomenon attributed to their short urethra and its proximity to the anal orifice^3^. Studies show that most UTI derive from gut bacteria, principally the gram negative family Enterobacterales^3^. Given the predictability of pathogens, empirical treatment has been traditionally adopted for uncomplicated UTI such as cystitis^2^. Among hospitalized patients, urinary catheterization often creates unnatural conditions that favor the successful establishment of a UTI. Such hospital-acquired infections including those associated with underlying co-morbidities (e.g., urinary tract abnormalities, immunosuppression, or pregnancy) are generally considered complicated UTI^1^. In such cases, the causative organisms tend to be more diverse and often difficult to predict. As such, complicated UTI cannot be managed empirically, requiring laboratory guided antibiotic selection^4^.

In developing countries such as Rwanda, where resource constraints frequently limit availability or access to routine laboratory diagnostic support, empirical treatment is common and has been found to be particularly convenient and cost-effective. Despite this advantage, empiric treatment tends to limit opportunity for temporal and spatial surveillance of antibiotic resistance. Even where causative pathogens are largely predictable, sensitivity profiles have been known to vary by geography and over time^5^. As such, guidelines for empiric treatment require regular review, to ensure their continued validity. In Rwanda, the national treatment guidelines recommend the use of nitrofurantoin or ciprofloxacin for empiric treatment of uncomplicated UTI^6^. However, these guidelines are more than ten years old and to ensure their continued validity in our region, we set out to identify and describe the sensitivity profile of uropathogens isolated at a regional hospital in northern Rwanda.

## Materials and methods

### Study design

This was a hospital-based, descriptive, and cross-sectional study employing quantitative methods of data collection and analysis. All symptomatic patients (≥ 13 years) referred to the laboratory for urine investigation were conveniently invited to take part. (Considering that sexual activity increases the risk of UTIs among females, and that age at sexual debut can be as young as 13 years in many African communities, the IRB approved the enrollment of such participants, who were treated as emancipated minors). A standard pre-designed tool was used to collect demographic, medical history, and clinical data. Participants provided a cleancatch midstream urine sample which was processed and analyzed.

### Study setting

The study was carried out at Butaro district hospital (BDH), a regional center of care located in Burera district, northern Rwanda. The hospital serves a community catchment of approximately five hundred thousand people, largely consisting of peasants engaged in crop farming with minimal involvement in commercial livestock activities^7^. The hospital provides in-patient and out-patient care in four major clinical disciplines namely internal medicine, pediatrics, general surgery as well as obstetrics and gynecology. At the time of the study, laboratory services included basic cytology, clinical chemistry, and histopathology. Notably lacking were microbiology services including routine cultures and drug sensitivity studies. Hence, for this study, these services were performed at the University of Global Health Equity (UGHE), a new institution offering medical education within the region. In addition, the hospital lacked an antimicrobial stewardship committee to guide rational antibiotic selection. Consequently, physician prescriptions rely heavily on clinical acumen as well as national standard treatment guidelines for common disease conditions. All services at the hospital are provided through a national health insurance scheme often with subsidized out-of-pocket cost to clients.

### Collection of socio-demographic data and urine samples

Nurses and laboratory technicians were trained to serve as research assistants. They were responsible for inviting and consenting prospective participants, as well as collecting sociodemographic data on a standard interviewer-administered tool (Appendix I). They were also trained on how to instruct and guide participants in the collection of a mid-stream (clean catch) urine sample. As most participants were not fluent in the English language, research assistants were required to be fluent in the local Kinyarwanda dialect so as to enable effective communication and instruction. Following proper hand washing, participants were led into a well-lit toilet facility and handed a sterile, well-labelled, wide mouth urine container (50ml) and accordingly instructed. Labelling consisted of participant's study number, sex and date of urine collection. Briefly on the collection method, the uncircumcised male was required to retract the foreskin on the glans penis prior to urine collection. He was required to void the first bit of urine into the toilet pan before collecting the mid-portion (starting approximately 2 seconds from beginning of urine flow) and then voiding the last bit again into the pan. He was then required to carefully close the container and deliver it to the research assistant for storage. In the case of female participants, they were required to wipe the urethral orifice (from front to back) with moist sterile gauze (dipped in normal saline) before collecting midstream urine as described for the males. All samples were stored under refrigeration (4°C) and transported to the UGHE laboratory within three hours of collection.

### Determination of true UTI and identification of uropathogens

In the laboratory, samples were registered and processed with each participant's study number serving as their unique identifier. The consideration of a sample as true UTI was arrived at using a two-stage screening process involving dip-stick urinalysis (qualitative analysis) followed by quantitative urine culture. This approach was adopted because neither approach alone, was considered sufficient to label a case as “true UTI”. For instance, urinary schistosomiasis or prior antibiotic use were each respectively, likely to confound findings from the above methods. For dip-stick urinalysis, a sample had to be positive for either leukocytes (µ+) or nitrites before it was further processed for culture in the second stage. For culture, a 1 µL sterile loop was used to pick and plate a well-mixed urine sample on both Mac-Conkey and blood agar, prepared according to standard methods (8). The use of these two media was aimed at optimizing the isolation of all possible organisms as well as using their unique growth characteristics to aid identification. All plates were inverted and incubated at 35°C (µ1) for 18-24 hours. A cutoff point of 105 CFU/ml was considered significant growth if the culture was pure and thus considered as “true UTI”. In addition, samples that were positive by urine dipstick but had less than 105 CFU/ml were considered true UTI if there was a positive history of antibiotic use prior to presenting at the hospital. Qualitative tests used in pathogen identification included unique colony growth characteristics, gram staining as well as standard biochemical tests, such as sugar fermentation using TSI agar, citrate utilization, indole production, the methyl red test, Voges Proskauer, urease, catalase, and coagulase tests, as well as novobiocin sensitivity for differentiating Staphylococcus. All tests were performed according to standard methods described elsewhere^8^.

### Antibiotic sensitivity assays

Antibiotic susceptibility tests were done on Mueller-Hinton agar (Sigma Aldrich, St. Louis, MO) according to the Kirby-Bauer method modified in accordance with the current guidelines from the European Committee on Antimicrobial Susceptibility Testing (EUCAST) (9). The following commercially prepared antibiotic discs (Oxoid Ltd., Cambridge, UK) were used: nitrofurantoin (100 µg), ciprofloxacin (5 µg), levofloxacin (5 µg), amoxicillin (10 µg), amoxicillin/clavulanate (20/10 µg), piperacillin/tazobactum (30/6 µg), cefuroxime (30 µg ), ceftriaxone (30 µg), Cefixime (5 µg), cefoxitin (30 µg ), cotrimoxazole (1.25/23.75 µg), trimethoprim (5 µg), gentamicin (10 µg), amikacin (30 µg), azithromycin (15 µg), fosfomycin (200 µg), meropenem (10 µg) and aztreonam (30 µg). These antibiotics and respective disc strengths were purposefully chosen with pharmacokinetic and pharmacoeconomic considerations suited to the treatment of UTI in resource-constrained settings. Measurements of inhibition zones were done to the nearest millimeter and interpreted according to EU-CAST guidelines^9^. Quality control of antibiotic susceptibility studies was performed once weekly using Escherichia coli ATCC 25922 and Staphylococcus aureus ATCC 29213 strains.

### Data entry and analysis

Laboratory observations were entered in participants' data file each day. Data was cleaned and regularly checked for completeness during the study. Each measurement of inhibition zone diameter was interpreted as ‘sensitive=S’, ‘sensitive with increased exposure=I’, or ‘resistant=R’ according to the most current EU-CAST standard interpretative charts^9^. At end of sample collection, data was double entered into Microsoft Excel- computer program and validated prior to analysis. The program was used to generate data summaries using standard descriptive statistics along with simple proportions among related variables.

### Ethical considerations

The study received ethical approval from the UGHE Research and Ethics Committee (Ref: UGHEIRB/2021/016). Administrative clearance was also obtained from the Butaro Hospital director before initiating study activities. Furthermore, written informed consent was obtained from each participant prior to enrollment into the study.

## Results

Over the 8 months' study period (November 2021-June 2022), we examined 198 urine samples of which 107 met the criteria for UTI by dip-stick urinalysis. Of these, 94 yielded significant growth on culture. Three urine samples yielded mixed growths. Among the 13 samples that did not yield any growth despite dip-stick positivity, 11 samples came from participants with a history of antibiotic use prior to visiting the hospital. One sample (from a male) did not yield any growth on both MacConkey and blood agar, despite presence of pyuria. Gram staining determined it as a possible case of gonococcus. Table 1 below provides a summary of the demographic and clinical characteristics of participants with UTI in the study. The most frequent presenting symptoms were dysuria and urge incontinence/increased urine frequency. Approximately thirteen percent (n=12) of participants (all female), presented with what were possibly recurrent UTI episodes. Thirty-five percent (n=33) of the study participants had used an antibiotic prior to hospital visit. The most common antibiotics used were amoxycillin (n=21), metronidazole (n=7), ciprofloxacin (n=5) and doxycycline (n=1).

**Table 1:** Demographic and clinical characteristics of patients presenting with urinary tract infections at Butaro district hospital

	Males (n=9)	Females (n=85)	Total
Mean age in years (range)	53 (39-60)	25 (13-73)	
Departmental origin of study participants			
Outpatient department	6	52	58
Internal medicine	2	9	11
Oncology	0	5	5
Surgery	1	0	1
Obstetrics/gynecology ^a^	n/a	18	18
Emergency department	0	1	1
Presenting clinical characteristics			
Dysuria	9	61	70
Suprapubic pain/discomfort	3	23	26
Urge incontinence/increased urine frequency		55	62
History of recurrent symptoms (in past 6 months)		12	12
Antibiotic use prior to hospital visit	3	8	11
Recent catheterization	1	3	4
Immune suppression symptoms	0	0	0
Known neurological/bladder disorders	0	0	0
Renal angle pain/tenderness	0	2	2
Prostatic enlargement	3	n/a	3
Positive urine sugar	2	3	5
Significant leukocyturia (pyuria)	9	85	94
Urine nitrite positive	3	75	78

Key: n/a, not applicable

a all samples came from pregnant women

### Diversity and proportions of uropathogens isolated

The diversity and relative proportions of uropathogens isolated is summarized in Table 2. Majority of organisms isolated were gram negative aerobes with Klebsiella pneumoniae contributing 35.1% (n=33) of the organisms, closely followed by Escherichia coli at 31.9% (n=30). Staphylococcal species were the third highest uropathogens contributing 20.2% of the total (n=19). These included Staph. saprophyticus, Staph. epidermidis and Staph. aureus with frequencies of occurrence being n=12, 5 and 2 respectively. All cases of Staph. epidermidis and Staph. aureus were isolated from female participants, except for one case of Staph. aureus from a catheterized male. One case of Salmonella spp was conclusively identified as a uropathogen.

**Table 2:** Diversity and susceptibility profile of uropathogens isolated at Butaro district hospital, northern Rwanda (n=94)

Uropathogens	Escherichia n=30			*Klebsiella pneumoniae* n=33			*Enterobacter spp* n=6			Proteus spp n=5			Salmonella spp n=1 Staphylococcus			

Antibiotic discs	S	I	R	S	I	R	S	I	R	S	I	R	S	I	R	S
Nitrofurantoin (100µg)	25	n/a	5	21	0	12	5	n/a	1	2	n/a	3	1	0	0	15
Ciprofloxacin (5µg	13	2	15	18	2	13	5	n/a	1	0	0	5	1	0	0	0
Levofloxacin (5µg)	11	6	13	12	5	16	3	2	1	0	0	5	0	0	1	0
Cefuroxime (30µg)	19	n/a	11	23	n/a	10	-	-	-	2	n/a	3	1	n/a	0	10
Ceftriaxone (30µg)	7	12	11	7	8	18	-	-	-	0	0	5	0	1	0	7
Cefixime (5µg)	16	n/a	14	11	n/a	22	-	-	-	0	n/a	5	0	0	1	-
Cefoxitin (30µg)	21	n/a	9	18	n/a	15	-	-	-	1	n/a	4	0	0	1	10
Gentamicin (10µg)	8	n/a	22	23	n/a	10	4	0	2	1	n/a	4	0	0	1	5
Amikacin (30µg)	17	n/a	13	22	n/a	11	4	0	2	3	0	2	1	0	0	8
Cotrimoxazole (1.25/23.75µg)	7	0	23	7	2	24	0	0	6	0	0	5	0	0	1	3
Trimethoprim (5µg)	8	n/a	22	11	n/a	22	0	0	6	0	n/a	5	0	0	1	4
Amoxycillin (10µg)	5	n/a	25	0	n/a	33	1	0	5	0	0	5	0	0	1	2
Amoxycillinclavulanate (20/10µg)	20	5	5	23	2	8	5	1	0	2	0	3	1	0	0	15
Piperacillintazobactum (30/6µg)	25	3	2	29	1	3	6	0	0	5	0	0	1	0	0	16
Azithromycin (15µg)	-	-	-	-	-	-	-	-	-	-	-	-	-	-	-	7
Fosfomycin (200µg)	6	n/a	24	3	n/a	30	-	-	-	0	n/a	5	0	0	1	-
Meropenem (10µg)	25	2	3	19	4	10	3	3	-	1	1	3	0	1	0	12
Aztreonam (30µg)	12	0	18	2	3	28	-	-	-	0	0	5	0	1	0	-

Key: n/a=not applicable, used where classification did not include the intermediate category, as per EUCAST.

Note: a dashed line appears in a cell where an antibiotic was not tested i.e., not recommended for that organism, as per EUCAST

### Antibiotic susceptibility and resistance profiles

For antibiotic susceptibility assays, the zone diameters for all quality control strains were within published limits. Table 2 shows the breakdown performance of each antibiotic against individual organisms. Clearly, resistance was observed against all antibiotics studied. To gauge suitability of study drugs for empiric therapy of community-acquired UTI, we merged frequency data from the categories ‘S=sensitive’ and ‘I=sensitive; increased exposure’ to show overall proportion of susceptibility versus resistance of each antibiotic against all organisms (Table 3). From an epidemiologic standpoint, a drug was considered suitable for empiric therapy if prevalence of resistance was less than 30%^10^. Thus, the best performing drugs were Piperacillin/tazobactum (8.5%), Amoxycillin/clavulanate (21.3%), Meropenem (24.5%), Nitrofurantoin (26.6%) and Cefoxitin (28%). In contrast, exceptionally high resistance levels were observed against amoxycillin (84%), cotrimoxazole (80%), trimethoprim (76%) and fosfomycin (87%). Furthermore, at a prevalence of 74%, gram negative organisms showed high resistance against aztreonam.

**Table 3:** Relative proportions of susceptibility and resistance from all uropathogens isolated at Butaro regional hospital, northern Rwanda (n=94)

Antibiotic (disc strength)	Sensitive	Sensitive, increased exposure	Total sensitive * (%)	Resistant (%)
Nitrofurantoin (100µg)	69	n/a	69 (73.4)	25 (26.6)
Ciprofloxacin (5µg)	37	8	45 (47.9)	49 (52.1)
Levofloxacin (5µg	26	21	47 (50.0)	47 (50.0)
Cefuroxime^a^ (30µg)	55	n/a	55 (62.5)	33 (37.5)
Ceftriaxone^a^ (30µg)	21	21	42 (47.7)	46 (52.3)
Cefixime^b^ (30µg)	27	n/a	27 (39.1)	42 (60.9)
Cefoxitin^a^ (30µg)	50	n/a	50 (72.4)	38 (27.6)
Gentamicin (10µg)	41	n/a	41 (43.6)	53 (56.4)
Amikacin (30µg)	55	n/a	55 (58.5)	39 (41.5)
Cotrimoxazole (1.25/23/75µg)	17	2	19 (20.2)	75 (79.8)
Trimethoprim (5µg)	23	n/a	23 (24.5)	71 (75.5)
Amoxycillin (10µg)	15	n/a	15 (15.9)	79 (84.0)
Amoxycillinclavulanate (20/10µg)	66	8	74 (78.7)	20 (21.3)
			
Piperacillintazobactum (30/6µg)	82	4	86 (91.5)	8 (8.5)
Fosfomycin^b^ (200µg)	9	n/a	9 (13.0)	60 (87.0)
Meropenem (10µg)	60	11	71 (75.5)	23 (24.5)
Aztreonam^b^ (30µg)	14	4	18 (26.1)	51 (73.9)

Key:

* Refers to sum of values in column 2 and 3. n/a: category not applicable as per EUCAST

a not tested against Enterobacter spp (n=6) as per EUCAST

b not tested against Enterobacter & Staphylococcus spp (n=25) as per EUCAST

## Discussion

Our study demonstrated considerable burden of antibiotic resistance in this largely rural community. Urinary tract infections are a common condition for which antibiotic use is often justified, accounting for a significant proportion of both outpatient and in-patient antibiotic consumption. Our findings did not differ from the many others that have identified the family Enterobacteriales as a major source of uropathogens^11^–^14^. In addition to UTI, Enterobacteriales are frequently implicated in life-threatening infections including pneumonia, endocarditis, pyelonephritis, septicemia, and surgical wound infections^15^,^16^. More worrisome perhaps, is the notoriety and ease with which Enterobacteriales share mobile genetic elements among themselves^17^. Given this background, antibiotic resistance patterns observed in this study have a direct bearing on treatment of other serious systemic infections. We found unacceptably high resistance frequencies (over 70%) against amoxycillin, cotrimoxazole, and trimethoprim, drugs that have long been heavily consumed in our setting. Similar frequencies were previously reported by Ntirenganya et.al., at a large referral hospital in the capital, Kigali^13^. The practice of self-medication with antibiotics has previously been reported among many rural and urban communities in Africa^18^. It comes as no surprise therefore, that 28% of our participants admitted to antibiotic selfmedication prior to visiting the hospital on this occasion. This practice has a direct effect on the selection and spread of antibiotic resistance. In a previous African study, the most common reasons cited for self-medication were; the need to cut costs and the need to act expeditiously to treat suspected bacterial infections^19^. Furthermore, the high frequency of resistance to antifolates may have been contributed to by their widespread use as prophylaxis against HIV-related opportunistic infections or even malaria in pregnancy. A surprising finding, however, was the equally high frequencies of resistance against fosfomycin and aztreonam, yet these drugs have rarely been used in our setting. Interestingly, more than 10 years ago, the study by Ntirenganya et al., using data from two large referral hospitals showed low resistance frequency against fosfomycin (approx. 9.7%)^13^. Such a change may be attributed to the efficient horizontal transfer of resistance factors among Enterobacteriales as previously described^17^.

Resistance to fluoroquinolones, aminoglycosides and cephalosporins generally ranged in the 40-60% levels. These frequencies are quite high and probably reflect the increase in consumption of drugs from these classes. More specifically, recent hospital data indicates high prescription rates for ciprofloxacin, gentamicin, and ceftriaxone in our setting. While planning this study, we purposively included possible alternatives from each of these classes (i.e., levofloxacin, amikacin and cefixime), in the hope that resistance to them would be less, given their limited community exposure. Unfortunately, resistance frequencies appear to be similar (Table 3), suggesting a high level of cross-resistance within each particular drug class. In contrast, two previous studies from Rwanda and Sudan reported significantly lower frequencies of resistance to amikacin (2% & 1.9%), where gentamicin resistance was high (37% & 35%, respectively)^13^,^20^. We suggest that the genetic mechanisms underpinning the two phenotypes are possibly different. Overall, these findings underscore the need for hospital formularies guided by locally generated antibiograms. Considering a 30% threshold for resistance^10^,^21^, none of the drugs from these classes are suitable for empirical therapy in our setting.

The lowest frequencies of resistance were observed with meropenem and the potentiated penicillins. Over 91% of organisms were sensitive to piperacillin-tazobactam, probably due to its limited exposure in our setting. Trends at our hospital show a rising consumption of amoxicillin-clavulanic acid and meropenem over the past ten years. It is however reassuring that approximately 78% and 75% of organisms respectively, remain sensitive to these drugs. An important clue to this slower rate of resistance selection/rise may be the fact that use of these drugs is largely limited to the hospital setting where they tend to be reserved for more serious infections. However, the selection and rise in carbapenemase producing Enterobacteriales is a matter of global concern^22^–^24^. A recent review on the epidemiology of carbapenem resistance within the East African region identified Klebsiella pneumoniae and E.coli as the most prevalent reservoirs^25^. Given the high burden of infectious diseases in our setting, measures to improve antibiotic stewardship are urgently needed. Our hospital is in the process of setting up a diagnostic microbiology laboratory which will hopefully, improve antibiotic selection practices. At 73% sensitivity, nitrofurantoin retained its effectiveness against most uropathogens. While this is reassuring, the drug can only be used for cystitis given its limited systemic reach.

At 35% (n=33), Klebsiella spp contributed the highest proportion of uropathogens in this study. This finding is quite perplexing as it is unlike many studies in the literature. It is however well-known that Klebsiella spp are generally resistant to penicillins such as amoxycillin, that lack beta-lactamase *inhibitor* cover. This was true in our study as none of the organisms was sensitive to amoxycillin (Table 2). Given this observation, communities that overuse or misuse such penicillins may inadvertently promote the proliferation of Klebsiella organisms in ways likely to risk superinfection with it. A similar phenomenon was reported in Taiwan, where, patients who had recently received amoxycillin or ampicillin were shown to have an increased risk of Klebsiella pneumoniae liver abscess^26^. We posit that a similar phenomenon may explain our present findings. An important strength of our study was the stringent two-stage criteria used to distinguish ‘true-UTI’ from asymptomatic bacteriuria or urine sample contamination. This approach enabled us to exclude many participants with lower abdominal symptoms due to other illnesses. We also attempted to stratify antibiotic susceptibility analysis based on risk differences (e.g., outpatient vs in-patients, or recurrent vs new infections). However, we did not find any trends or significant differences between such groups. This may have been due to the relatively small dataset that lacked the power to detect such differences. Given this limitation, a decision was taken to omit such stratification from the results in order to avoid concerns about validity. Furthermore, it is recommended that in order to ensure statistical validity of hospital antibiograms, they should consist of no less than 30 organisms, preferably obtained from the same body site^27^. Our data was able to achieve this for the two major organisms of UTI burden i.e., *E. coli* and Klebsiella pneumoniae. We are, therefore, confident that this offers validity and generalizability of our findings to the study population. It also follows that data for the other organisms may not be a true reflection of their sensitivity profile, given the low numbers. Nevertheless, the data serves as an early indication of sensitivity trends, providing a base on which we continue to build a comprehensive database for future informed decision making.

## Conclusion and recommendations

Our study revealed unacceptably high frequencies of resistance to amoxycillin, cotrimoxazole, trimethoprim, fluoroquinolones, aminoglycosides and cephalosporins, all of which exceeded 50%. Resistance to the potentiated penicillins, meropenem, cefoxitin and nitrofurantoin were less than 30%. To curb the rising burden of antibiotic resistance, community as well as hospital interventions towards better antibiotic use are urgently needed. More importantly, microbiology services to guide rational antibiotic selection are particularly important and long overdue. For the empirical treatment of community-acquired UTI in this part of Rwanda, nitrofurantoin remains effective whereas fluroquinolones are not.
